# The Future of Scientific Leadership is Interdisciplinary: The 2019 CAS Future Leaders Share Their Vision

**DOI:** 10.1016/j.isci.2020.101442

**Published:** 2020-08-20

**Authors:** Jovana V. Milić, Andreas Ehnbom, Mahlet Garedew, Paulette Vincent-Ruz, Tracy H. Schloemer, Gregory K. Hodgson, Meagan S. Oakley, Koichi Sasaki, Subhash Chander, Marc-André Légaré, Cassandra E. Callmann, Aisha N. Bismillah, Dannie J.G.P. van Osch, Vanessa Sanchez, Nathan R.B. Boase, Dickson Mambwe, Connor W. Coley, Yuanxin Deng, Kerry N. Betz, Jesús Sanjosé-Orduna, Sean Natoli, Liang Zhang, Olga Bakulina, Ehsan Fereyduni, Jazmín Ciciolil Hilario-Martínez, Lucas Busta, Arianne Hunter, Yoonsu Park, Farnaz Haidar Zadeh

**Figure:**
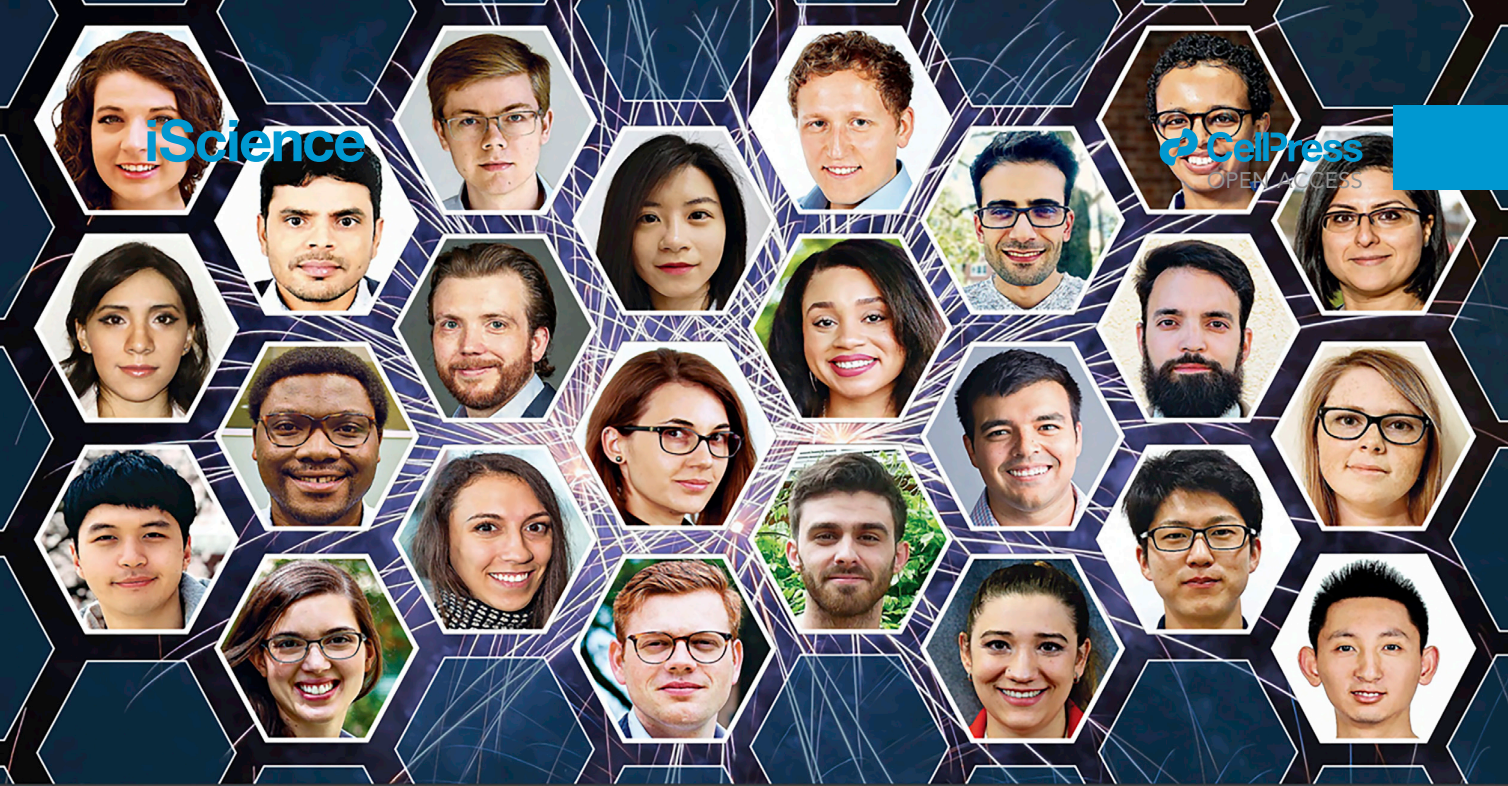
CAS Future Leaders 2019 (Photo credit: Liz Neeley, The Story Collider)

For the last decade, the CAS Future Leaders program has gathered early-career scientists from across the globe based on their outstanding accomplishments in the field of chemistry to provide support to participants in cultivating their own voices and futures in scientific leadership. The goal of the program has been to empower early-career scientists like us to begin to shape our own future leadership roles, from learning to convey effective speech by developing our own research stories to growing to be better mentors for the next generation of future leaders. In 2019, to honor the 10th anniversary of the program, the CAS Future Leaders program encompassed essential leadership skills divided into five topics, namely, *storytelling*, *insights*, *strategies*, *perspectives*, and *impact*, some of which were new to the program this year. However, what was not new to the program was an emphasis on the potential *global* impact that this program could make. To do this, the program brought together in this cohort 29 post-docs and graduate students, from 16 countries. A staple of this program is not only the breadth of countries that are represented but also the many facets of chemistry that are represented as demonstrated later in the article. One reason for this is that a leader in the sciences will need to be open to innovations across discipline and geographical boundaries, something that we explored a lot during our time together.

After months of anticipation, excitement, and email exchanges leading up to the program, we finally met in person in downtown Columbus, Ohio. We spent our days learning and conversing about leadership in science and what leadership looks like in each of our experiences, whereas in the evenings we had the opportunity to network. Whether it was over dinner or game night, where we bonded over a friendly ping-pong and duckpin bowling, or going to the nearby North Market to experience the diverse food options, the time we spent at the program was carefully designed to facilitate both formal and informal networking and dialogue. The weeklong training in Columbus was followed by additional networking opportunities at the ACS National Meeting and Exposition, which took place in San Diego, California. While often conferences can be overwhelming, especially for early-career researchers, the training that we had and the connections we had fostered already made this and future networking opportunities even more tangible. Now after a decade of this program around 200 participants are part of what has become a *“CAS Future Leaders Family.*” After all, this program has never been only about leadership training but rather lasting friendships, personal and professional support and a community that is the product of bringing a diverse group of scientists together to stimulate resonating conversations. While the program has left us with long-lasting memories, knowledge, and transferrable skills to use for the rest of our careers in science, it has also left us with the urge to share our discussions with a broader audience and confidence to attempt global action.

As we reflect on what we have learned from this program, apart from skills to enhance our research skills and communication, we ask ourselves: *what does the future of science leadership look like?*

To answer this and try to understand our vision from the perspective of the diverse, multidisciplinary background to which this cohort belongs, we focused on the following three questions.

(1) What do you find to be the key scientific challenges over the next decade?
(2) What is the role of chemists in addressing some of these contemporary challenges?
(3) What are the essential components for science leadership?

Here, we highlight summaries of our answers to each question and present a selection of responses (answers from all participants can be found in the **Supplemental Information**).

**Figure:**
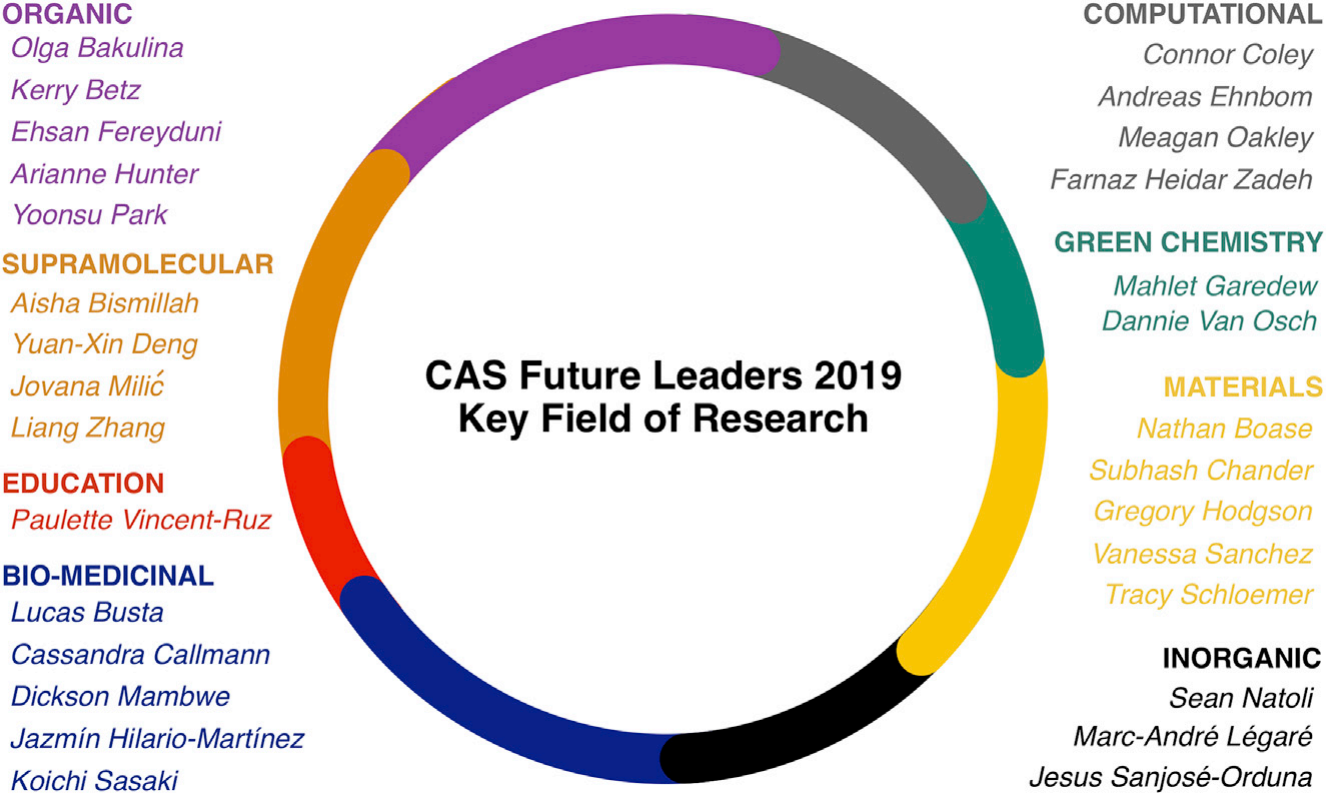
An approximate division of the CAS Future Leaders 2019 by their respective area of expertise in the research field of chemistry.

## Contemporary Challenges

What do you find to be the key scientific challenges over the next decade?

As leaders we recognize the importance of having a vision rooted in awareness and an understanding of the most pressing problems, both in our respective areas of expertise and global challenges. Participation in this program inspired discussions, reflections, and actions on several of the most crucial global challenges, such as sustainability, climate change, human health, the equitable access to science for underrepresented groups, and raising awareness of systemic issues of gender inequality and mental health in academia.

**Paulette:** One of the biggest challenges we face as scientists is to educate and train an ever-increasing diverse population without the tools to do so. Higher education institutions and research culture is built to keep marginalized people away; how do we successfully make these changes to diversify our ranks?

**Arianne:** Gone are the days where scientists could hide behind books, papers, and journals and their findings be respected by the general public simply because they are a “scientist.” Scientists now have the challenge of properly communicating their work if they desire it to have a broader impact and change the world we live in.

**Farnaz:** Thinking about the next decade, my mind is flooded by the many challenges we are facing, all of which have a scientific component and need to be accompanied by (inter)national education, policies, planning, etc., to increase the impact of scientific discoveries.

**Yuanxin:** There is always a trade-off between simplicity of synthesis and complexity of functions in chemistry. In the past decade, many functional complex matters have been developed to improve the properties and cater to market demand. But most materials suffer from high-cost complex preparation, which limits their application. It is really essential to develop functional materials with really low cost and high performance.

**Cassandra:** The synthesis of safe and effective drug delivery systems continues to be a key scientific challenge. As our population continues to age, this problem is becoming more and more significant. Thus, developing a molecular understanding of the complex interactions between synthetic materials and biological systems is critical. This understanding can then be used to develop new biomaterials that are highly specific to their intended biological target, opening the door for new ways to fight disease. This will require the collaboration of scientists from a multitude of disciplines and from diverse backgrounds, including academia, industry, and government.

**Nathan:** Waste, and in particular plastic waste, is a huge challenge the world is facing at the moment, and is quite controversial. At the end of the 20th century polymers and plastics facilitated the cheap supply of single-use, short lifetime goods. While this is not sustainable, we cannot underestimate the role that these materials have played in improving the quality of our lives, and a more equitable supply of goods. The chemical and process technologies developed for these bulk commodity goods have laid the foundations for the development of advanced polymeric materials for use in future applications in energy generation, energy storage, fighting disease, and smart materials to improve our everyday lives. Not all plastics are “bad.”

**Vanessa:** Especially in the context of our aging population, we must provide technology that enables people of different abilities and incomes to live independently and equitably. As future leaders, we are part of the generation that witnessed the information age change the world, when computers and the internet broke down barriers to access to education, connectedness, and global understanding; a similar revolution in assistive technology is on the horizon.

**Koichi**: It is now obvious that our activities are not sustainable at all. Research in the fields of chemistry, materials engineering, and synthetic biology may play central roles in providing breakthroughs for minimizing environmental burdens regarding production of energies, foods, and materials.

**Yoonsu:** I believe one of the key challenges in chemistry is to develop new plastics, which can be readily degradable in an on-demand manner. Commercial plastics in our age are literally destroying our planet even at this moment, but we do not have a clear solution for the situation.

**Gregory:** In my opinion, the 2020s will challenge scientists to make great strides toward sustainability and science diplomacy. The global scientific community must collectively respond to climate change, global health crises, underrepresentation, and inequity in order to succeed in leaving the world a better place than we found it.

## The Role of Chemists

What is the role of chemists in addressing some of these challenges?

**Figure:**
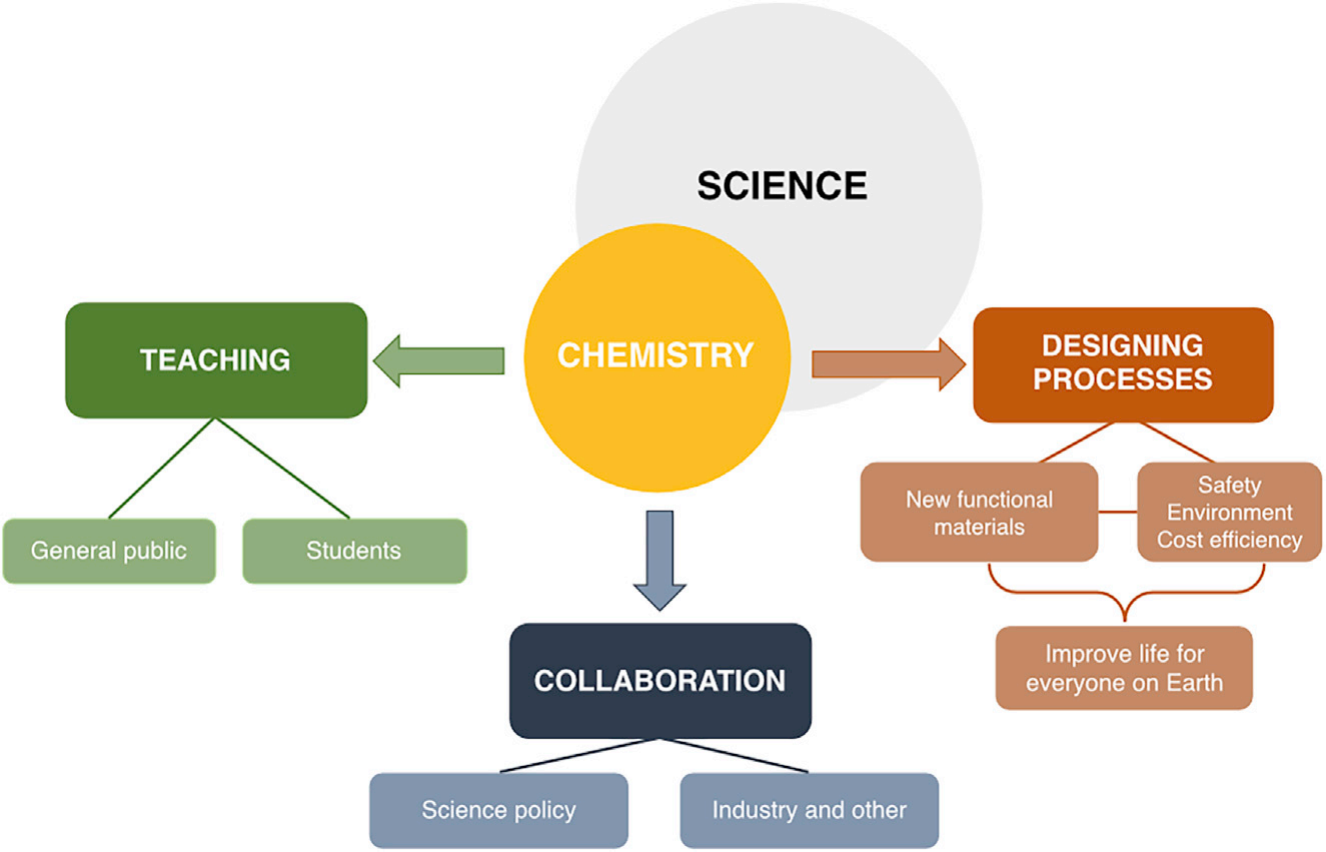
A visual representation of the responses provided to the question on the role of chemists in addressing some of the contemporary challenges. Full responses are provided in the Supplemental Information.

On the question regarding our role as scientists and chemists, we all seem to recognize that we hold the responsibility of being the forward thinkers of our time in three main areas, namely, (1) education, (2) designing safer and cost-effective processes when exploring new chemical transformations, and (3) engaging in collaborative efforts, bridging gaps between disciplines, and reaching out to both related and unrelated fields, such as policy-making and economics. These major themes regarding our role as future leaders in science are addressed in more detail below and have further inspired our own future application within our own respective disciplines.

**Jovana:** Addressing some of the contemporary challenges requires an interdisciplinary approach, and chemists can play an instrumental role at the interface of multiple disciplines by designing and developing functional materials, as well as advocating for science and policies.

**Tracy:** Chemists play one key role in addressing sustainable practices, but not the only role. We must partner with many researchers outside of our “silos” to meet these ends: from materials scientists, physicists, engineers, and business leaders.

**Meagan:** Chemists have a huge role to play in equality and equity issues, especially those in leadership positions. Far too often we focus on only the chemistry, and we forget about the chemists behind it all. Step out of your comfort zone and educate yourself on equality issues, and most importantly, take the time to listen to your students and colleagues for the betterment of our workplace. We can achieve all of these key challenges and more when everyone has a seat at the table.

**Olga:** The very idea of personalized medicine involves the use of interdisciplinary research, in which chemistry plays a leading role. The development of specialized therapeutic agents will not be possible without contribution of synthetic chemists, biochemists, and computational chemists.

**Kerry:** Chemistry can be a wasteful science, as every organic chemist has seen, with disposable plastic syringes, waste chemicals, and energy-intensive reactions - these are only a few of the everyday ways an organic chemist impacts the world. On a larger scale, chemists aim to create new reactions and ways of transforming common chemicals into valuable targets; reducing waste, cost, and energy in these methods is a significant concern. Organic chemists should be conscious of the wasteful way their science can impact the world and strive to design the most efficient and economical reactions possible.

**Ehsan:** Do not obsess with numbers. Numbers of publications, higher impact factors, etc. These days, it is difficult to draw a line between disciplines. Therefore, I believe that chemists along with other disciplines should work tighter together on the world's most pressing problems.

**Sean:** One of the defining pillars of this scientific era is the ever-growing spirit of collaboration. Monumental research is accomplished by bringing together a team of scientists from multiple disciplines each leveraging their expertise to solve long-standing challenges in science. As chemists, we contribute our fundamental perspective on atomic interactions to the scientific equation.

**Liang:** Nowadays, the term "chemist" is becoming quite a broad definition as lots of “chemists” are working in highly overlapping areas. So, in my view, besides utilizing our scientific talents/abilities, what we could contribute more to make it come true is to establish proper collaborations and set up an efficient platform in which everyone could add their talent to the challenges.

## Call to Action

What are the essential components for science leadership in your opinion?

Finally, to make an impact and address these contemporary challenges, we must lead the change we wish to see in the world and the essential components of this science leadership are identified as follows. The themes that were most prevalent were effective communication, collaboration, as well as creativity and passion, and we hope to actively practice these skills, while attempting to bring them out in others as we hope other mentors will try to do as well.

**Figure:**
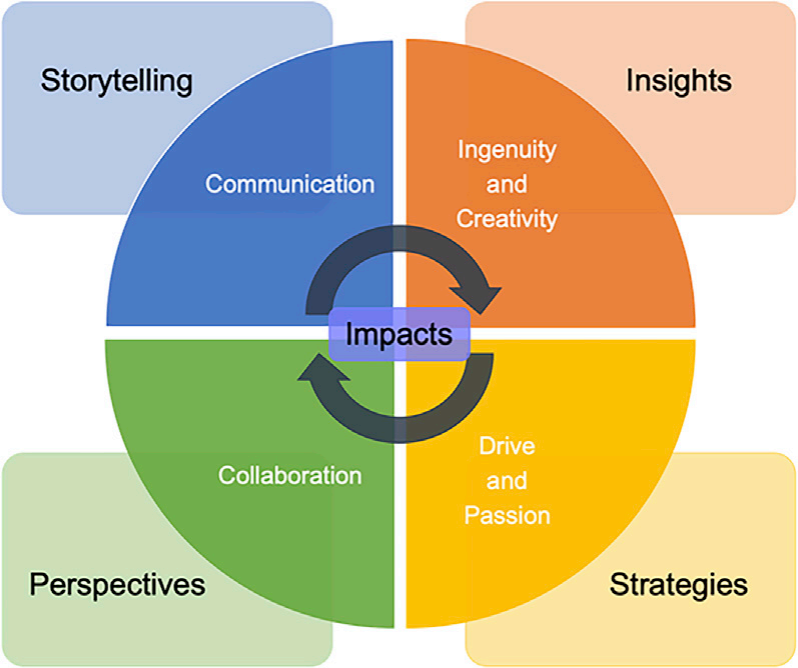
A visual representation of our vision of the essential components of science leadership underpinned by the themes of the 2019 CAS Future Leaders program.

**Andreas:** I think scientific leadership should be rich in collaboration and innovation, but most importantly a leader needs to have a challenge-seeking character that enjoys discovering new things, even in times of hardship to overcome obstacles, i.e., be resilient. I find it tremendously important that you try to uplift students/colleagues working around you to elevate the entire scientific community and to not be afraid of discussing problems.

**Mahlet:** First, scientific leaders should have the willingness to have a collaborative, cross- and interdisciplinary mindset as well as the willingness to see the problems through varying lenses. Second, having a systems-thinking approach and the willingness to work at the boundaries and intersections of different systems, I believe, is instrumental. Science leadership should push these boundaries to enable new advances. Finally, science leadership should also have an aspect of mentorship and outreach to bring diverse perspectives, to bridge the gap between scientific and non-scientific communities, and to make the knowledge accessible to all.

**Dannie:** Essential components in scientific leadership are the ability to listen to other people, motivate the people you work with, and a tremendous motivation to learn new things.

**Subhash:** Scientists should be able to persuasively clarify their research, be capable of distinguishing themselves, and communicating boldly regarding their research findings to other scientists/non-scientists. A scientist can become a good science leader by joining a professional society and getting involved, seeking opportunities to collaborate, and being proactive.

**Lucas**: I believe a leader should cultivate the ability to bring balance to the efforts of which they are a part. This means, for example, prioritizing self-sufficiency and critical thinking in their mentees while still providing some concrete direction, incorporating new technologies and protocols into their research while not neglecting the benefits of established routines, and being critical about new ideas in their field but also open to evidence that supports new views and directions.

**Aisha:** Communication, confidence, and courage! Communication to ensure that you can clearly connect and inspire others within science, confidence to accomplish high goals and lead powerfully, and courage to harness new ideas while taking the steps that no one else has yet.

**Connor:** One of the most important components of scientific leadership to me is openness in communication. Openness to the ideas of others; to sharing successes and failures with trainees; to sharing the limitations, implications, and assumptions of new discoveries with the research community, policymakers, and the public; and to two-way feedback and change.

**Jazmín:** Education. To promote professional development, to produce very qualified researchers. To promote active multidisciplinary collaboration to address current science challenges. To invest and promote cutting-edge science research projects.

**Dickson:** Science leadership should go beyond technical mastery or the ability to secure funding for research. I honestly think it begins at having the ability to clearly separate the project, from the team (who are people). Some of the essentials include being a good listener, admitting to mistakes, engaging with co-workers at all levels, appreciation of work environment dynamics, and the ability to handle conflict both tactfully and decisively.

**Jesús:** We must leave behind the cliché of the “mad scientist” in the basement doing investigations alone. If we want to really be part of the change, we have to start embracing the idea of cooperativity between fields and collaborate with our partners in the borders of our scientific field.

**Marc-André:** The leaders who will help solve our key scientific challenges will be the people who can work with and inspire others to work for the benefit of all. They will inspire their peers and a new generation with their ideas and questions and will enable others to solve problems.

We hope that this program, and this snapshot of our own vision of the future, inspires other scientists and young professionals to partake and embrace their vision for a better world by translating their own ideas into global action. We are excited to contribute to these efforts and hope that you will continue to follow our journeys, using our story as a source of inspiration while creating your own.

## Supplemental Information

Document S1. Additional Interview Responses

