# Supplementary material for: The Future of Scientific Leadership is Interdisciplinary: The 2019 CAS Future Leaders Share Their Vision
Source: iScience. 2020 Aug 20;23(9):101442. doi: 10.1016/j.isci.2020.101442 (PMC7520891; doi:10.1016/j.isci.2020.101442)
Supplement: Document S1. Additional Interview Responses [file mmc1.pdf]

iScience, Volume *23*

Supplemental Information

The Future of Scientific Leadership is Interdisciplinary: The 2019 CAS Future Leaders Share Their Vision

Jovana V. Milić, Andreas Ehnbom, Mahlet Garedew, Paulette Vincent-Ruz, Tracy H. Schloemer, Gregory K. Hodgson, Meagan S. Oakley, Koichi Sasaki, Subhash Chander, Marc-André Légaré, Cassandra E. Callmann, Aisha N. Bismillah, Dannie J.G.P. van Osch, Vanessa Sanchez, Nathan R.B. Boase, Dickson Mambwe, Connor W. Coley, Yuanxin Deng, Kerry N. Betz, Jesús Sanjosé-Orduna, Sean Natoli, Liang Zhang, Olga Bakulina, Ehsan Fereyduni, Jazmín Ciciolil Hilario-Martínez, Lucas Busta, Arianne Hunter, Yoonsu Park, and Farnaz Haidar Zadeh

Supplemental Information

The Future of Scientific Leadership is Interdisciplinary: The 2019 CAS Future Leaders Share Their Vision

Jovana V. Milić,* Andreas Ehnbom,* Mahlet Garedew,* Paulette Vincent-Ruz, Tracy H. Schloemer, Gregory K. Hodgson, Meagan S. Oakley, Koichi Sasaki, Subhash Chander, Marc-André Légaré, Cassandra E. Callmann, Aisha N. Bismillah, Dannie J. G. P. van Osch, Vanessa Sanchez, Nathan R. B. Boase, Dickson Mambwe, Connor W. Coley, Yuanxin Deng, Kerry N. Betz, Jesús Sanjosé-Orduna, Sean Natoli, Liang Zhang, Olga Bakulina, Ehsan Fereyduni, Jazmín Ciciolil Hilario-Martínez, Lucas Busta, Arianne Hunter, Yoonsu Park, Farnaz Haidar Zadeh

Correspondence email: jovana.v.milic@gmail.com, lehnbom@tamu.edu, mahlet.garedew@yale.edu

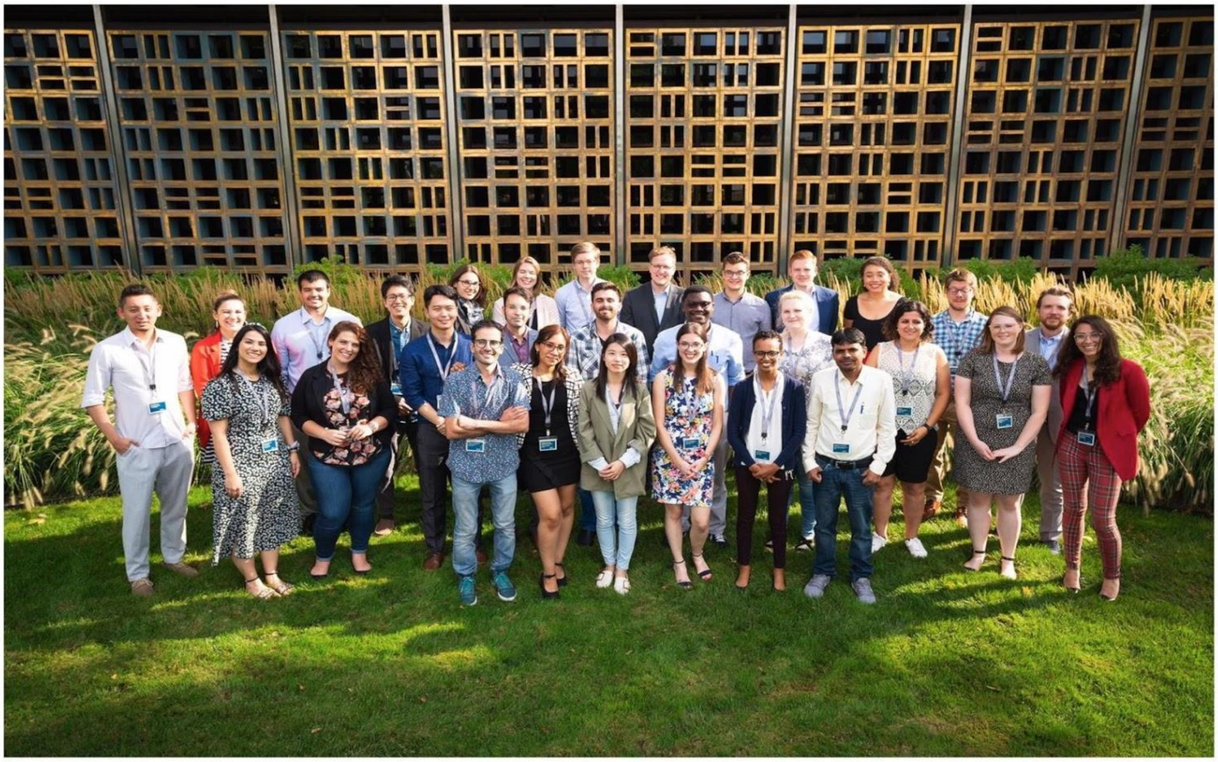

CAS Future Leaders 2019 (Photo credit: CAS)

(1) What do you find to be the key scientific challenges/developments over the next decade?

Jovana: I believe that some of the key scientific challenges over the next decade will involve the strive towards sustainability in terms of resources, technologies and global awareness. To this end, interdisciplinary collaborations will play a critical role in addressing these challenges, with the increasing importance of the development of new materials and processes. This progress should also involve greater involvement of the community in science advocacy and policy making.

Andreas: Generally speaking, I think there are a lot of urgent challenges in the area of green technology and energy, which must be addressed if our society is going to continue to be prosperous. In the area of computational chemistry, I think there will be tremendous growth as we observe what can be done when experimental chemists meet computers and theory. As many fields become interdisciplinary, I think the organic chemistry community will talk more with biochemists in the development of future catalysts. I'm excited to follow the development of synthesis guided or assisted by AI/machine learning technology. This field is something I believe has the potential to dominate and catalyze progress in all fields and sub disciplines of chemistry, not only classical synthetic chemistry disciplines (organic/inorganic).

Mahlet: I believe enabling greener and sustainable technologies that can effectively displace/replace human dependence on fossil-derived energy and chemicals continues to be one key challenge. Though many advances have been achieved in the past few decades in the area of renewable technologies, the crucial questions of high capital costs, lack of infrastructure, and inefficiency of various renewable technologies remain the topics of interest in today's scientific community. Specifically, in the realm of a bio-based energy and chemical manufacture, integrated biorefineries that are efficient in atom and energy input and outputs, I believe, will continue to be the target in the next decade.

Paulette: One of the biggest challenges we face as scientists is to educate and train an ever- increasing diverse population without the tools to do so. Higher education institutions and research culture is built to keep marginalized people away; how do we successfully make these changes to diversify our ranks?

Tracy: I see bridging technology from the academic to industry as a tremendous challenge and opportunity to work towards. In light of climate change, this is especially pressing as our society grapples with the question "What does it mean to be sustainable while maintaining/lifting all to a high quality of life (e.g., plastics for packaging/sanitation, etc.)?" For instance, translating recent developments in academia in polymer recycling to scalable, viable technologies will be a tremendous challenge both technology- and funding-wise.

Gregory: In my opinion, the 2020's will challenge scientists to make great strides toward sustainability and science diplomacy. The global scientific community must collectively respond to climate change, global health crises, underrepresentation and inequity in order to succeed in leaving the world a better place than we found it.

Meagan: I believe over the next decade there needs to be a shift in academic culture. The endless number of articles reporting on sexual harassment, gender inequalities, mental health issues (and more) stemming from treatment in the academy show that there are many problems, but provide few solutions. Many leaders are already promoting more inclusive teaching and working environments, hopefully providing a nucleation site for growth as a society.

Koichi: It is now obvious that our activities are not sustainable at all. Research in the fields of chemistry, materials engineering and synthetic biology may play central roles in providing breakthroughs for minimizing environmental burdens regarding production of energies, foods, and materials. Otherwise, we will eventually have to focus on aerospace engineering to find a different place to live.

Subhash: As per my concern, the breakthroughs will come to action on climate change and the transformation of renewable energy systems. For this, we will have policies that can accelerate the progress to act on the climate. While the transformation of renewable energy systems will be well on track, innovative approaches and technologies will have to develop which allow us to achieve our goals faster.

Cassandra: Some of the key scientific challenges facing the world over the next decade include antibiotic resistance, as well as increased water scarcity as a consequence of climate change. These are global health challenges whose solutions will require the collaboration of scientists from a multitude of disciplines and from diverse backgrounds, including academia, industry, and government.

Aisha: I believe that one of the key scientific challenges that we will face over the next decade will be to use the research, i.e., the fundamental knowledge that we are gaining, within real-life applications. The question we need to constantly be asking ourselves is: how can we use our novel research to solve the pressing challenges that society is currently faced with.

Marc-André: Over the next decade, the most important challenges are centered on the efficient and sustainable use and reuse of natural resources and of energy.

Dannie: When considering the major (scientific) challenges in the world, we have to ensure the replacement of our fossil resources with renewable ones. Moreover, we have to avoid the major pollution of plastics by recycling or by using alternative starting materials, such as bio-based ones. Both challenges have to be addressed both from the scientific and commercial point of view. The time for this is now and not tomorrow!

Nathan: Waste, and in particular plastic waste, is a huge challenge the world is facing at the moment, and is quite controversial. At the end of the 20th century polymers and plastics facilitated the cheap supply of single use, short lifetime goods. While this is not-sustainable, we cannot underestimate the role that these materials have played in improving the quality of our lives, and a more equitable supply of goods. The chemical and process technologies developed for these bulk commodity goods have laid the foundations for the development of advanced polymeric materials for use in future applications in energy generation, energy storage, fighting disease and smart materials to improve our everyday lives. Not all plastics are “bad”.

Dickson: The paradigms of artificial intelligence (AI) in drug discovery have continued to progress and we’ve seen the critical role this technology has played. It is therefore no surprise that it is in this era, where de novo lead generation, a process that ideally takes nearly two (2) years, could be done in only 46 days, a case of In silico Medicine and the GENTRL platform. With more AI solutions companies on the rise, as well as increased research in an academic group setting, I can predict a reduced timeline for not only the drug research process, but also lower pre-clinical drug attrition rates due to efficient data mining and better accuracy of predictions. Additionally, emerging trends in automation and miniaturization in chemistry, enabling rapid synthesis and screening of drug libraries will translate into more diversity of discovery pipelines by delivering high quality starting points for research.

Vanessa: Especially in the context of our aging population, we must provide technology that enables people of different abilities and incomes to live independently and equitably. As future leaders, we are part of the generation that witnessed the information age change the world, when computers and the internet broke down barriers to access to education, connectedness, and global understanding; a similar revolution in assistive technology is on the horizon.

Connor: Two major categories of challenges that require the most immediate attention, in my opinion, are human health and climate change. Climate change poses an existential threat and will continue to do so unless we dramatically change the resources we use for energy, transportation, agriculture, and manufacturing. At the same time, emerging diseases, pandemics, and biological threats like antibiotic resistance cannot be ignored; access to affordable medicines is essential for maintaining a high quality of life.

Yuanxin: There is always a trade-off between synthesis simplicity and function complexity in chemistry. In the past decade, many functional complex matters have been developed to improve the properties and cater to market demand. But most materials suffer from high-cost complex preparation, which limits their application. It is really essential to develop functional materials with really low-cost and high-performance.

Kerry: I believe that climate change is one of the biggest challenges that scientists, policy makers, individuals, and nations will face in the next few decades. Already we are seeing the devastating effects of human-caused climate change and feeling the economic and social impacts of these events. I believe that science and technology development will play a key role in how we respond to climate change over the next decade. New sources of energy, different ways of disposing of waste, and technology that enables us to interact with the world in new, green ways are just a few of the key areas in which I envision scientists can have enormous impact.

Sean: Green Chemistry. One of the greatest challenges facing today's society is global waste. Therefore, as scientists we should push to discover efficient, eco-friendly, and sustainable methods for creating many of the materials and products we use today.

Jesús: Without any doubt, one of the more critical challenges we are going to face, as society, in the next decade, is the rational and efficient use of natural resources. The development of a sustainable society, in all its aspects, is going to be crucial, in order to avoid compromising the ability of future generations to meet their own needs.

Liang: I am working on the area between organic chemistry and supramolecular chemistry, which means I am quite interested in building up molecules and establishing the connection between functions and structures. Therefore, I think there are two key challenges that lay on the boundaries: 1) How could AI assist the development of new synthetic toolbox? Would it be possible to achieve atom-precise-synthesis by activating any bonds we want and attach any functional group on any position of the backbone we wish? If so, it would be a revolution for synthetic methodology and we could make any compounds as we designed, draw the platform of functionalities and connect those to structural characters. 2) Could we precisely control the self-assembly process, particularly in asymmetrical self-assembly process, apart making giant/complex molecules in one port, how could we achieve the stepwise self-assembly process, introduce different building blocks in any sequential matter and understand the consequences of resulted assembly generating from subtle changes of conditions (like structures, solvent, anion, cation etc.)?

Olga: The development of gene therapy and personalized medicine is one of these key challenges, since health improvement concerns absolutely every person. Two of my family members are struggling with diabetes and they have great hopes and interest for this kind of research.

Jazmín: I consider that the central challenge for the next decade involves the solution for the current alarming problems of actual society. One of the most important challenges for scientists is related to the current environmental problems like climate change and pollution. Scientists and most governments agree that the world faces an environmental crisis. Moreover, the development of a safe and effective detection and treatment of diverse diseases that are the leading causes of death like cancer, ischemic heart disease, respiratory diseases, Parkinson and Alzheimer. As the rate of aged people is increasing all over the world, their inherent muscle atrophy must be attended to improve their quality of life. Finally, due to the world population rapidly increasing, there is a need to guarantee the supply of food, this involves research in diverse areas in order to increase food production.

Ehsan: I think scientists have attached their success to ‘numbers’! The higher the number of publications, the more accepted they feel! The higher the impact factor, the happier they feel! Their efforts should pave the way for a better world not counting ‘numbers’! This is currently a challenge and will get worse in the future!

Lucas: The past decades have seen the development of incredibly information-rich databases in fields of both chemistry and biology. Connections between these databases have exceptional potential to foster and accelerate rational searches for new bioactive chemicals, inform the design of synthetic metabolic pathways, and substantially enhance our understanding of the genetic basis for natural product diversity. However, such connections are minimal at best, and there is a critical need for broadly generalizable tools that link chemical and genomic databases to help address grand challenges in chemical biology.

Arianne: I believe one of the key scientific challenges over the next decade will be scientists’ ability to communicate their knowledge with the rest of society in an efficient and impactful way. Effective science communication is a necessity if we hope to invoke necessary changes in society. Gone are the days where scientists could hide behind books, papers, and journals and their findings be respected by the general public simply because they are a “scientist”. Scientists now have the challenge of properly communicating their work if they desire it to have a broader impact and change the world we live in.

Yoonsu: I believe one of the key challenges in chemistry is to develop new plastics, which can be readily degradable in an on-demand manner. Commercial plastics in our age are literally destroying our planet even at this moment, but we do not have a clear solution for the situation.

Farnaz: Thinking about the next decade, my mind is flooded by the many challenges we are facing, all of which have a scientific component and need to be accompanied by (inter)national education, policies, planning, etc. to increase the impact of scientific discoveries. In my opinion, some of the more recent challenges, which require special attention, include: 1) Reducing our ecological footprint and using natural resources responsibly, 2) Integrating artificial intelligence technology in research and society and addressing its ethical dilemmas, and 3) Fulfilling open- science practices, as well as establishing and adhering to ethical conduct in research.

(2) What is the role of chemists in addressing some of these contemporary challenges?

Jovana: Addressing some of the contemporary challenges requires an interdisciplinary approach and chemists can play an instrumental role at the interface of multiple disciplines by designing and developing functional materials, as well as advocating for science and policies.

Andreas: I think that the chemistry education our current students (both on the graduate and undergraduate level) need to be upgraded to match the fast-paced development in chemistry. I think one key aspect, unless you have the capability to completely tailor your own education, is that chemistry programs with a certain "track" should include more programming in their curricula. The faster we can design and can keep up with our educational programs the larger leaps we will see in subsequent ripening of young talents.

Mahlet: As scientists we have an important role in addressing current global challenges by designing materials, products, processes, and systems intelligently and with intentionality for a greener future that benefits posterity and presents minimal harm to the environment. For this, it is crucial that we continue the work towards improving efficiency of processes, reduce process complexity and waste, and design for reduced toxicity of the processes, products, and overall systems. Our role is to be leaders in the shift that is needed in how we view the challenges and, in the way, we hope to address the most pressing questions.

Paulette: Chemists need to accept that RESEARCH = TEACHING. Without this framework change it will be impossible to keep up with the training and research demands of the world.

Tracy: Chemists play one key role in addressing sustainable practices, but not the only role. We must partner with many researchers outside of our "silos" to meet these ends: from materials scientists, physicists, engineers, and business leaders.

Gregory: Chemists can play a pivotal role in addressing these challenges by developing new functional materials while conscientiously striving toward sustainable chemical processes, and by becoming more effective at science advocacy and communication.

Meagan: Chemists have a huge role to play in equality and equity issues, especially those in leadership positions. Far too often we focus on only the chemistry, and we forget about the chemists behind it all. Step out of your comfort zone and educate yourself on equality issues, and most importantly, take the time to listen to your students and colleagues for the betterment of our workplace. We can achieve all of these key challenges and more when everyone has a seat at the table.

Koichi: Quite simply, we can directly contribute to realize necessary technological advances. Keep trying our best would be a great way to educate the next-generations.

Subhash: The key social and economic factors are directly and indirectly propelling climate changes and increasing energy demands, thus being a materials scientist (or any flavor of a chemist), the chemists play significant roles in addressing these challenges. Chemists share the responsibility to assist activities in relieving the causes of climate change and in transferring renewable energy systems by providing new materials and innovative processes since the common goal is to solve both problems. Beyond existing energy technologies, the perovskite-based solar cell technology will be a high potential next-generation low-cost and high-efficiency photovoltaic technology; consequently, it will be enormously useful for the world and society to cater the current energy demand.

Marc-André: Chemists have to and can contribute in a very real way. From the development of new materials for sustainable housing, to the valorization of waste materials, including greenhouse gases, to recycling, and to waste mitigation they are at the center of many crucial topics in terms of sustainable use of resources.

Cassandra: Chemists will have a central role in addressing all of these challenges. Indeed, a significant body of work already exists towards the development of novel antibacterial materials to overcome antibiotic resistance and new materials to trap water and other important life- sustaining molecules. Moving forward, chemists will be instrumental in developing new approaches for tackling these global challenges!

Aisha: To address the challenge of utilizing research within real-life applications, chemists need to walk hand-in-hand with collaborators in different fields, such as engineering for example, whilst working closely with industries.

Vanessa: Coming from a materials science perspective, I believe our role is to understand where each of us fits within science and engineering as a whole and to contribute our own expertise to collaborative efforts with researchers from other disciplines. This approach requires actively seeking discussions with researchers from different backgrounds and working through problems together.

Nathan: Beyond the obvious technological roles that chemists will play in researching new chemistries, new materials and improving processes for fabrication and manufacturing, they must also lead the way in communicating and educating the public about their science. We must be engaged in open and honest discussion with industry partners, end users and policy makers; ensuring that new materials improve our lives, without unintended consequences in waste generation or the safety of people and the environment. We can't remain ignorant of the ethical questions our science and research will create, hiding behind the veil of the "innocence of knowledge".

Dickson: Synthetic experimental design could essentially be done by AI, based on reactions that are already known, but much less can be said about the inventing ability of the technology in diverse chemical spaces, chemists must therefore continue to invent! It is important as a medicinal chemist to align and continue using tools like AI as assistants in simplifying processes that would otherwise not be accomplished in time for the benefit of a specific objective.

Connor: Chemistry and its adjacent disciplines are what drive innovation of new molecules, materials, and processes. The goals of certain subfields like medicinal chemistry and catalysis are perfectly aligned with these priorities.

Yuanxin: Chemists are working on fundamental research to explore the tremendous potential of chemistry. Many chemists are trying to explore the intrinsic tunability of molecules, utilizing the subtle and intriguing "intelligence" of molecular structure to enable their self-organization towards highly complex architectures. The reliability and diversity of this pathway have been proved by nature. The role of chemists should be the key "catalyst" to accelerate this evolution process.

Kerry: Chemistry can be a wasteful science, as every organic chemist has seen, with disposable plastic syringes, waste chemicals, and energy-intensive reactions only a few of the everyday ways an organic chemist impacts the world. On a larger scale, chemists aim to create new reactions and ways of transforming common chemicals into valuable targets; reducing waste, cost, and energy in these methods is a significant

concern. Organic chemists should be conscious of the wasteful way their science can impact the world and strive to design the most efficient and economical reactions possible.

Dannie: We as chemists and chemical engineers should take the lead and start working interdisciplinary to solve all these problems. Everywhere we look, fuels for transportation, materials of your bikes, all made possible with via chemistry. We only have one earth and it is the highest time to save it!

Sean: One of the defining pillars of this scientific era is the ever-growing spirit of collaboration. Monumental research is accomplished by bringing together a team of scientists from multiple disciplines each leveraging their expertise to solve long standing challenges in science. As chemists, we contribute our fundamental perspective on atomic interactions to the scientific equation.

Jesús: Chemists have an unrivalled opportunity to be part of the next paradigm shift. The understanding of how complex chemical transformations work is an extremely powerful approach for the development of more efficient and sustainable reactions. Through fundamental understanding of the mechanistic intricacies of these transformations, we, as scientists, might have an unparalleled opportunity to overcome the limitations of inefficient transformations, and also develop innovative processes by using rational design.

Liang: For the challenges I listed above, I would think chemists are only one piece of the blueprint. Also, nowadays, chemists are becoming quite a broad definition as lots of 'chemists' are working on the highly overlapping area. So, in my view, besides utilizing our scientific talents/abilities, what we could contribute more to make it come true is to establish proper collaboration, set up an efficient platform in which everyone could add their talent to the challenges, remove the political/regional/sexual/ethnic effects in science and focus on solving pure scientific problems. Meanwhile, it would be always good for chemists (but also for all other scientists) to believe that all inventions are neutral, and it just depends on how we use it. Moreover, we are more than 'a' scientist/chemist when we start working independently, another role we have is a supervisor. We should also pass the knowledge and experiences to the young generation and provide the soil and fertilizer that allows them to grow up quickly and properly.

Olga: The very idea of personalized medicine involves the use of interdisciplinary research, in which chemistry plays a leading role. The development of specialized therapeutic agents will not be possible without contribution of synthetic chemists, biochemists and computational chemists.

Jazmín: Owing to chemistry is a central science that supports and contributes to the development of many other disciplines, chemists play essential roles in the aforementioned research challenges: As climate change issues become more prevalent, there's a demand for chemists who can develop sustainable solutions like new biodegradable materials or biofuels; Chemists make important contributions to the discovery of safer drugs, vaccines and effective treatments. This is a growing need for the control of infectious disease that can greatly increase morbidity and mortality; In nanoscience, chemists are the key to the development of molecules with applications in crucial areas such as health, agriculture, nanotechnology, nanomedicine. Chemists play an essential role in order to obtain nanomaterials in an efficient, sustainable and cheaper way, on a large scale and with a low environmental impact.

Ehsan: Do not obsess with numbers. Numbers of publications, higher impact factors, etc. These days, it is difficult to draw a line between disciplines. Therefore, I believe that chemists along with other disciplines should work tighter together on the world's most pressing problems. Do not work to publish, solid work will get published...

Lucas: Chemists can help create links between chemistry and biology by applying their expertise with chemical structures and chemical informatics to advancing their capabilities with biological structures (DNA and RNA) and biological informatics. Tandem fundamental abilities in these areas will enable work that bridges these disciplines and their databases. My current research, as an example of such a pursuit, focuses on developing and evaluating two basic information- processing pipelines: (i) tools that retrieve and visualize the structural relationships between a group of natural chemicals and their distribution across species in the tree of life, and (ii) tools that correlate relationships between chemical structures with specific enzymatic transformations, and such enzymatic transformations with genetic information from the species in which those chemicals are found.

Arianne: I believe it is the job of chemists to view our science as more than a "basic physical science" that is difficult for the general public to understand. We must make an effort to explain our work in common terms and relay its importance effectively.

Yoonsu: Interdisciplinary effort might be necessary to deal with the plastic issue. For example, organic and inorganic chemists need to collaborate for better catalyst design, and material scientists' insight might be inevitable for engineering monomer and fine structures.

Farnaz: Chemistry focuses on characterizing the properties of matter and learning how to predict and explain the changes it undergoes. It is fundamental in solving many future problems, because most of the challenges we are facing, from drug discovery to waste-management and sustainable energy, require identifying novel molecules and materials with desirable properties. Experimental and theoretical chemistry, in combination with artificial intelligence and technological advances, have given us a deeper understanding of the interplay between structure and properties leading to faster discovery of new compounds.

(3) What are the essential components for science leadership in your opinion?

Jovana: The essential components for leadership in science are effective communication and collaboration with integrity and passion for problem-solving, shared vision, and project management, along with active listening and learning.

Andreas: I think scientific leadership should be rich in collaboration and innovation but most importantly a leader needs to have a challenge-seeking character that enjoys discovering new things, even in times of hardship to overcome obstacles, i.e. be resilient. I find it tremendously important that you try to uplift students/colleagues working around you to elevate the entire scientific community and to not be afraid of discussing problems.

Mahlet: First, scientific leaders should have the willingness to have a collaborative, cross- and interdisciplinary mindset as well as the willingness to see the problems through varying lenses. Second, having a systems-thinking approach and the willingness to work at the boundaries and intersections of different systems, I believe, is instrumental. Science leadership should push these boundaries to enable new advances. Finally, science leadership should also have an aspect of mentorship and outreach to bring diverse perspectives, to bridge the gap between scientific and non-scientific communities, and to make the knowledge accessible to all.

Paulette: People need to learn to listen, they need to be willing to accept they are wrong and EDUCATE themselves about how to become better mentors and teachers. Without this people may be effective publishers of research but not good leaders.

Tracy: From my perspective as an early career scientist, essential components to scientific leadership is making sure the job gets done well while contributing to an overall positive, collaborative working environment. This is complicated: sometimes it means doing things that "aren't my job", sometimes it means delegating, and sometimes it means engaging in challenging conversations with colleagues. A leader assumes the best intentions from others: everyone on a team cares and wants to do well. A leader helps bring the best out in others as well as from herself.

Gregory: For me, the most essential ingredients for effective science leadership are communication, perseverance, empathy and active listening.

Meagan: The most important quality in a scientific leader would be a passion for learning. I think the most effective leaders are those who are constantly reading new articles and attending seminars outside of their wheelhouse. This encompasses so many individual qualities like listening and having natural curiosity. Another quality I think is important is to be genuinely excited about research. It can become infectious to those around them and can provide the team with intrinsic motivation.

Koichi: The essential components for science leadership are integrity and passion.

Subhash: The training of science leadership plays a crucial role for a comprehensive career advancement plan. Being the CAS Future Leaders 2019, scientific leaders are required to push the amalgamation of science into the broader world in such a direction that sites science in a further prominent position to assist change the world for the betterment. Scientists should be able to persuasively clarify their research, be capable of distinguishing themselves, and communicating boldly regarding their research findings to other scientists/non-scientists. A scientist can become a good science leader by joining a professional society and getting involved, seeking opportunities to collaborate, and being proactive.

Marc-André: Essential components of science leadership include altruism, vision, open- mindedness, and willingness to learn from others. The leaders who will help solve our key scientific challenges will be the people who can work with and inspire others to work for the benefit of all. They will inspire their peers and a new generation with their ideas and questions and will enable others to solve problems.

Cassandra: An essential component of science leadership is an unwavering drive to answer the most significant scientific challenges, and, moreover, the ability to instill this same eagerness in those you mentor and collaborate with. Good communication skills are key in this regard!

Aisha: Communication, confidence and courage! Communication to ensure that you can clearly connect and inspire others within science, confidence to accomplish high goals and lead powerfully, and courage to harness new ideas while taking the steps that no one else has yet.

Nathan: Support and understanding are essential components for scientific leadership, particularly in the academic research sphere. The predominant model for academic research has involved a pyramid type structure, with a relatively few number of individuals building teams of junior researchers and students. Success in research has become so focused on ever increasing quantitative measures of “success”, including numbers of publications, or value of research grant income. We have lost the humanistic measure of the success of our roles: the education of students, the mentorship of emerging scientific leaders, the collaborations and collegiality. By being more supportive of those scientists that we are educating and mentoring, and measuring our value as human beings, we will be more effective leaders.

Vanessa: Two essential components for science leadership are creativity and empathy. Creativity allows leaders to find interesting and meaningful problems to tackle, and to generate new approaches when the research team hits an apparent roadblock. Empathy enables leaders to effectively mentor, guide, and train the next generation of scientists to continue exploring!

Dannie: Essential components in scientific leadership are the ability to listen to other people, motivate the people you work with, and a tremendous motivation to learn new things.

Dickson: Science leadership should go beyond technical mastery or the ability to secure funding for research. I honestly think it begins at having the ability to clearly separate the project, from the team (who are people). Some of the essentials include being a good listener, admitting to mistakes, engaging with co-workers at all levels, appreciation of work environment dynamics and the ability to handle conflict both tactfully and decisively.

Connor: One of the most important components of scientific leadership to me is openness in communication. Openness to the ideas of others; to sharing successes and failures with trainees; to sharing the limitations, implications, and assumptions of new discoveries with the research community, policymakers, and the public; and to two-way feedback and change.

Yuanxin: In my opinion, great sensitivity to the forefront of science is the most essential component for science leadership. Only by staying at the forefront of science can new things be created constantly. The other components for science leadership may be the courage to pursue innovation, a rigorous attitude towards science, excellent presentation and organization skills.

Kerry: Scientific leaders should above all seek to be educators and uplift those around them. A leader enables their team to accomplish its goals through support, training, advocacy, and a positive work environment. Too often I have encountered leaders who seek to inspire through intimidation, salesmanship, or harshness. A true leader is not driven by their own fame or success but by that of their teammates. Caring about the health and well-being of those one leads serves to inspire loyalty and creativity, and can only encourage and foster scientific thinking.

Sean: An attitude of purpose and a spirit of ingenuity. Advances in science require hours (sometimes decades) of failure in order to achieve success. Good leaders are passionate about solving challenging problems and pursue them with an unrivaled tenacity. Great leaders share those characteristics but additionally strive to continuously pioneer novel ways of approaching scientific problems, thereby inspiring others. The spirit of innovation is highly contagious, and leads to tremendous intellectual development and growth for all team members.

Jesús: We must leave behind the cliché of the mad scientist in the basement doing investigations alone. If we want to really be part of the change, we have to start embracing the idea of cooperativity between fields, and collaborate with our partners in the borders of our scientific field.

Liang: For the science leadership, I think the insight, responsibility, confidence and courage are the essential components. The insight will offer you the deep understandings in your area and guide the direction of your team, making a clear plan with fast decisions on each step of the development. It also could help you to discover the characters of each person in your team and organize the team in the best format. The responsibility is also quite important as the leader should take the consequences when the team makes mistakes, meanwhile, the responsibility also means the leader should be the example for the team and follow

the rules equally. The confidence and courage give the leader a good personality which will make the team compact and in total harmony.

Olga: I believe, science leadership requires passion for science, broad education, ability to "think globally and act locally" and the ability to inspire other people to do or support science.

Jazmín: Education. To promote professional development, to produce very qualified researchers. To promote active multidisciplinary collaboration to address current science challenges. To invest and promote cutting-edge science research projects.

Ehsan: In my opinion to be a great leader, we need to have communications and influencing skills, strong analytical, critical-thinking and problem-solving skills.

Lucas: I believe a leader should cultivate the ability to bring balance to the efforts of which they are a part. This means, for example, prioritizing self-sufficiency and critical thinking in their mentees while still providing some concrete direction, incorporating new technologies and protocols into their research while not neglecting the benefits of established routines, and being critical about new ideas in their field but also open to evidence that supports new views and directions.

Arianne: I believe 2 essential components for science leadership are "relatability" and determination. Relatability is important because anyone can be a great scientist that is stuck in the laboratory doing work but still be completely unrelatable because people don't interact with you. However, if you take the initiative to be relatable and get to know your co-workers and others in your field they are more apt to see your passion for the science and more willing to be led by you. Determination is important because science is a career path filled with failure and frustration, if you let the small thing deter you will never make it to the position to be a leader.

Yoonsu: Being open-minded towards other disciplines and continuous effort to apply new strategy for problem solving.

Farnaz: The best leaders are active learners and, in my opinion, some of the most essential components of scientific leadership are constructive communication, engagement, open- mindedness, mentorship, mutual respect, integrity, and mindfulness. These set the stage for a productive scientific dialog and foster creativity and collaboration. Unfortunately, leadership is one of the most overlooked components of scientific training exacerbated by the fact that 1) the community is mostly, if not only, focused on scientific output, and 2) leaders are most often not held accountable, and 3) there is an imbalance of power between leaders and team members. One potential solution is considering leadership as part of the evaluation processes (i.e. by grant agencies, universities, etc.) by collecting feedback from team members, similarly to how we gather students' feedback as part of a course evaluation to assess an instructor's performance.
